# Human liver organoid: modeling liver steatosis and beyond

**DOI:** 10.1186/s13619-023-00161-y

**Published:** 2023-04-03

**Authors:** Jinsong Wei, Wen Zhang, Bing Zhao

**Affiliations:** 1 State Key Laboratory of Genetic Engineering, School of Life Sciences, Zhongshan Hospital, Fudan University, 200438 Shanghai, China; 2 Greater Bay Area Institute of Precision Medicine (Guangzhou), Fudan University, Nansha District, 511458 Guangzhou, China; 3 Institute of Organoid Technology, bioGenous Biotechnology, Inc, 215125 Suzhou, China

## Abstract

Steatosis, as the early stage of nonalcoholic fatty acid disease (NAFLD), would progress into nonalcoholic steatohepatitis (NASH) and liver failure without intervention. Despite the development of animal models, there is still a lack of the human-relevant platform for steatosis modeling and drug & target discovery. Hendriks et al., reporting in *Nature Biotechnology*, leveraged human fetal liver organoids to recapitulate steatosis by introducing nutritional and genetic triggers. Using these engineered liver organoid-derived steatosis models, they screened drugs that alleviate steatosis, and mined common mechanism of effective compounds. Further, inspired by the results of drug screening, the arrayed CRISPR-LOF screening targeting 35 lipid metabolism genes was performed, and FADS2 was identified as a critical regulator of steatosis.

## Main text

**Fig. 1:**
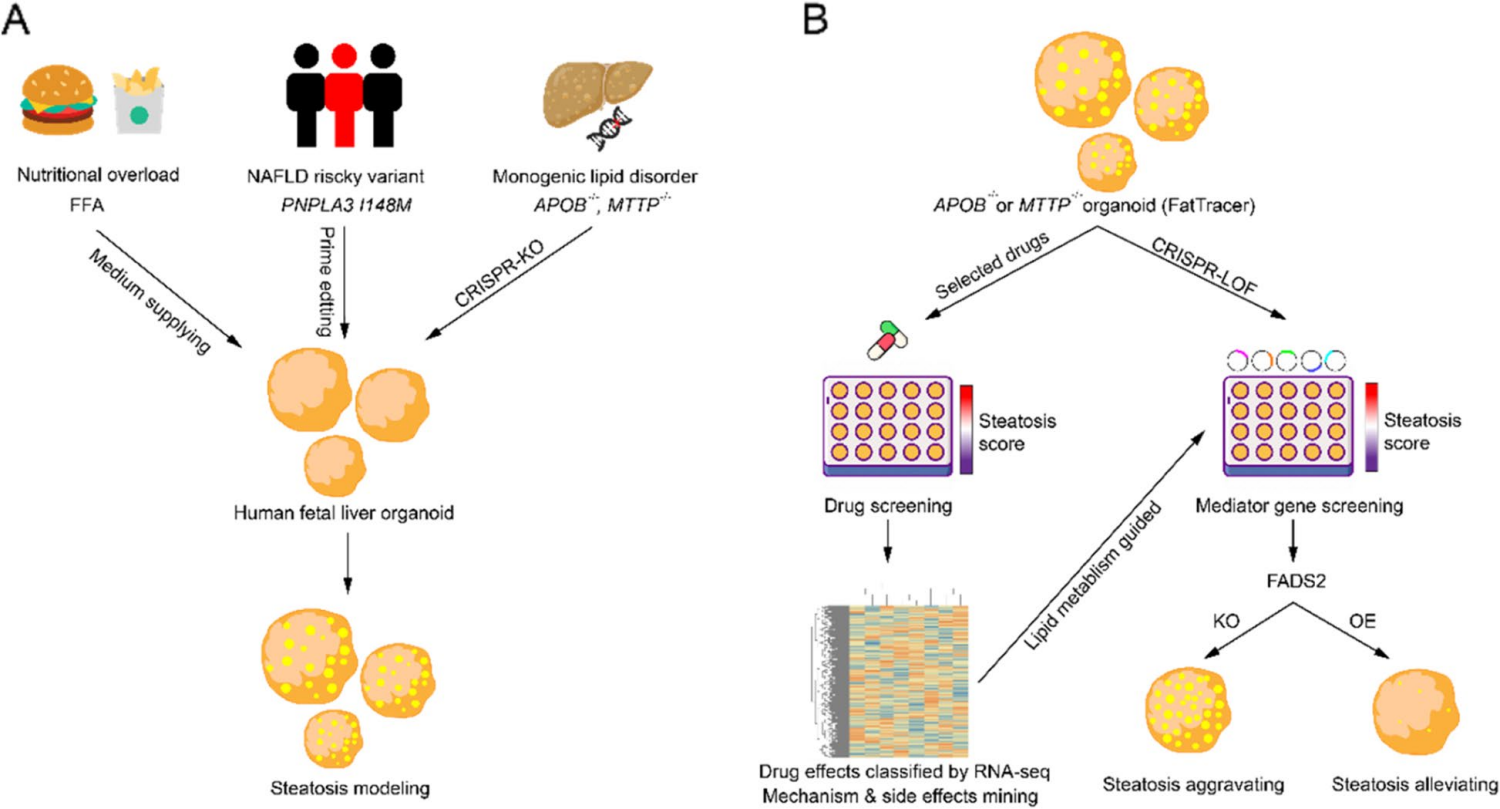
Establishing steatosis model with human fetal liver organoid and screening the drugs/targets with steatosis reducing effect. **A** Schematic of steatosis modeling with human fetal liver organoid by introducing variant triggers. **B** Schematic of drug screening coupled RNA-seq analysis and CRISPR-LOF screening with subsequent functional validation

Nonalcoholic fatty liver disease (NAFLD) has emerged as “Silent Killer” for a quarter of the world’s population. Starting from steatosis, which is featured by triglyceride (TAG) accumulation in the hepatocytes, it would aggravate into steatohepatitis, liver fibrosis and liver cancer or failure. Therefore, blocking NAFLD at steatosis stage is critical. The main causes of NAFLD are high-calorie diets and predisposed genetic mutations. To mimic those triggers, western diet fed and risky gene mutated animal models were developed to facilitate mechanism investigation and drug discovery. However, FDA-approved drugs for NAFLD is not available on the market till now, raising the question that whether previous models could fully recapitulate the pathophysiological features of human NAFLD. Organoids derived from human tissue faithfully document the cell lineages and behaviors of their origin, and have been used to model organ development (Liang et al. 2022), regeneration (Wei et al. 2019), tumorigenesis (Yang et al. 2022) and virus infection (Zhao et al. 2020). Different from iPCS derived liver organoids that adopted step-wised differentiation protocols, human liver tissue derived organoids could proliferate and be subjected to screening to address gene’s functions (Wei et al. 2019; Yang et al. 2022). Hendriks et al. (Hendriks et al. 2023) utilize expandable human fetal liver organoid to mimic the steatosis under various triggers, and further proved its promising applications by performing drug screening and CRISPR-LOF screening (Fig. 1).

Steatosis is caused by three main triggers: overload nutritional diet, high risky variant (PNPLA3 I148M) and predisposed monogenic lipid disorders (APOB or MTTP mutation) (Fig. 1A). Hendriks et al. decided to model those triggers one by one, then investigate their interactions. Firstly, they supplemented the cultures of human fetal organoids with free fatty acids (FFA), in order to approximate the western diets. Ideally, high concentrated FFA exposure led to lipid accumulation in organoid, which closely resembled hepatocyte steatosis. Visualized by Nile Red staining, the degree of steatosis could be quantified and scored. Then they used CRISPR tools to generate different PNPLA3 mutations in organoids: PNPLA3 I148M^−/+^ (HE), PNPLA3 I148M^−/−^ (HO) and PNPLA3-KO. Compared with WT organoids, all of those variants resulted in steatosis: PNPLA3-KO yield highest steatosis score, followed by HO and HE. Notably, Pnpla3-KO did not cause steatosis phenotype in mice model, highlighting the interspecies difference in disease modeling. They also found the PNPLA3 I148M^−/−^ mutation aggravated the FFA induced steatosis in organoid, indicating the PNPLA3 I148M^−/−^ might be more susceptible to high fat diet induced NAFLD. The last trigger they modeled was the mutations of *APOB* or *MTTP*, which are involved in *de novo* lipogenesis (DNL) and very low density lipoprotein (VLDL) secretory. As expected, the engineered human fetal liver organoids carrying *APOB*^*−/−*^ *or MTTP*^*−/−*^ showed significant intracellular lipid accumulation. [U-^13^ C]-glucose isotope-tracing also demonstrated the DNL contribution to the lipid accumulation in *APOB*^*−/−*^ *or MTTP*^*−/−*^organoids.

After establishing variant steatosis organoid models, Hendriks et al. set out to perform drug screening to hunt steatosis resolving compounds (Fig. 1B). Using steatosis score as the readout, they scanned a panel of anti-NAFLD drugs (positive on animal models), and found inhibitors of ACC, FAS and DGAT2, a FXR agonist, as well as recombinant hFGF19 could efficiently alleviate the steatosis in FFA models and *APOB*^*−/−*^ *or MTTP*^*−/−*^ models. However, other anti-NAFLD drugs, such as PPAR agonists, thyroid receptor-beta agonist and sirtuin 1 activator failed to do so, which might be explained by their targeting stages (more advanced NAFLD), cell lineages (immune cells), and the most likely, the interspecies bias. In turn, those results also suggested that the steatosis models developed by Hendriks et al. were purely hepatocyte autonomous and sensitive to DNL relative drugs, which is crucial for subsequent new target screening. It is still superficial to merely assess the steatosis-reducing effect of those drugs, Hendriks et al. then probed the underlying mechanisms by interrogating transcriptomic profiles. By analyzing the RNA-seq data of drug-treated organoids, they classified the drugs into 3 different groups based on the DEG clustering, which implied convergent mechanism of suppressing DNL would be effective to treat steatosis. Meanwhile, the side effect of drugs, such as proliferation impairment and epithelial-to-mesenchymal transition have been underscored.

Inspired by the drug screening results, Hendriks et al. decided to hunt new mediators of steatosis in organoids (Fig. 1B). Previously established *APOB*^*−/−*^ *or MTTP*^*−/−*^organoids exhibited severe steatosis phenotype, and could be passaged for long-term expansion. So they renamed it FatTracer, on which the arrayed CRISPR-LOF screening was performed to target DNL related genes. The positive controls (ACCi, DAG2i and PLNP3-KO), which have been validated in previous drug screening, consistently reduced the steatosis in this FatTracer platform. After interrogating 35 candidate genes, they found the perturbation of FADS2, an enzyme mediating the rate-limiting step in the biosynthesis of polyunsaturated fatty acids (PUFAs), aggravated steatosis in FatTracer. Further validation by knockout FADS2 in WT organoids also phenocopied steatosis. Interestingly, overexpression FADS2 alleviated steatosis in both FatTracer and FFA induced models. To entangle the underlying mechanism of FADS2 in reducing steatosis, Hendriks et al. analyzed lipidomics of FADS2^WT^, FADS2^−/−^ and FADS2^OE^ organoids, and found the FADS2^OE^ reduced total TAG content and metabolized from newly synthesis short chain TAGs to long chain PUFAs. However, it is unclear to what extent long chain PUFAs have beneficial effects on steatosis.

## Conclusions

Hendriks et al. developed variant steatosis models using human liver fetal organoids. Being exposed to free fatty acids (approximating nutritional overload) and genetic manipulation (mimicking NAFLD risky mutations), the human liver organoids well recapitulate steatosis. Beyond modeling, the authors further performed drug screening and genetic screening, to re-evaluate the existing anti-NAFLD drugs in human-relative organoid models, as well as to discover new target for resolving steatosis. During drug screening tethered in-depth transcriptomic analysis, they claimed repressing DNL would be an effective way to reduce steatosis. During CRISPR-LOF screening on FatTracer platform and subsequent functional characterization, they identified FADS2 as a critical mediator for steatosis.

In fact, liver steatosis modeling, including nutritional overload or risky genetic mutation, had been done by other groups using iPSC derived spheroid/organoid (Ouchi et al. 2019; Pingitore et al. 2019; Tilson et al. 2021). However, limited by the proliferative capacity, those models could not be applied to larger scale drug/gene screening. Hendriks et al. filled the gap by harnessing the inherently proliferative properties of human liver organoid derived from fetal tissues. Therefore, combined with state-of-art gene editing techniques, the expandable organoids are no longer merely modeling tools, but open the huge revenue for the development of drugs & therapies. In additional to these novel achievements, their study also raises several open questions: Steatosis primarily occur in adult stage, to what extent the immature liver fetal organoid could mimic steatosis? FatTracer relied on relative rough bright field imaging of intracellular lipid droplets, could any lipid-specific nanoprobe be applied to achieve precise and real-time quantification of steatosis? Lipid accumulation is a relatively late hallmark of steatosis, could single-cell CRISPR screening be applied to FatTracer for early steatosis mediator hunting? Hendriks et al. demonstrated the potential of human liver organoids empowered by CRISPR tools, pointing the trend of cross-disciplines in the field of organoid technology.

## Data Availability

Not applicable.
